# Coverage, Formulary Restrictions, and Out-of-Pocket Costs for Sodium-Glucose Cotransporter 2 Inhibitors and Glucagon-Like Peptide 1 Receptor Agonists in the Medicare Part D Program

**DOI:** 10.1001/jamanetworkopen.2020.20969

**Published:** 2020-10-15

**Authors:** Jing Luo, Robert Feldman, Scott D. Rothenberger, Inmaculada Hernandez, Walid F. Gellad

**Affiliations:** 1 Division of General Internal Medicine, Department of Medicine, University of Pittsburgh School of Medicine, University of Pittsburgh, Pennsylvania; 2 Department of Pharmacy and Therapeutics, University of Pittsburgh School of Pharmacy, University of Pittsburgh

## Abstract

This cross-sectional study assesses drug coverage, formulary restrictions, median retail prices, and annual out-of-pocket costs associated with commonly used insulin products across Medicare Part D plans.

## Introduction

The Centers for Medicare & Medicaid Services recently announced a voluntary plan to cap out-of-pocket costs associated with insulin products in participating enhanced Part D plans.^1^ However, this model will not apply to other high-cost glucose-lowering medications such as sodium-glucose cotransporter 2 (SGLT2) inhibitors and glucagon-like peptide 1 (GLP-1) receptor agonists. These classes are increasingly used as second-line agents for patients with type 2 diabetes despite only a modest effect on glycemic control (approximately 0.8% to 1%) because of mounting evidence of cardiovascular benefits. We sought to examine contemporary coverage and out-of-pocket costs for beneficiaries filling either an SGLT2 inhibitor or GLP-1 receptor agonist prescription in Medicare Part D.

## Methods

This cross-sectional study used the 2019 quarter 1 Prescription Drug Plan Formulary, Pharmacy Network, and Pricing Information Files^2,3,4,5^ to assess drug coverage, formulary restrictions, median retail prices, and annual out-of-pocket costs associated with commonly used SGLT2 inhibitors and GLP-1 receptor agonists (Table) across Part D plans (both stand-alone and Medicare Advantage). Because each drug is available in several different formulations and package sizes, we report estimates for only the most commonly dispensed National Drug Code according to Medicaid State Drug Utilization Data for 2019 and that represented the drug label’s recommended maintenance dose. We excluded combination products because they are infrequently used. We calculated the percentage of Part D plans that covered each drug without formulary restrictions, defined as having no prior authorization and no step therapy requirements. We also calculated the median retail price and interquartile range (IQR) for a 30-day supply for each drug. We reported the percentage of plans that covered each drug at tiers 1 to 3 (ie, as a preferred brand-name drug or better). All estimates were weighted by average plan enrollment during quarter 1 of 2019. Plans with enrollment less than 10 were excluded.

**Table.** Coverage, Formulary Restrictions, and Retail Prices for SGLT2 Inhibitors and GLP-1 Receptor Agonists Across Medicare Part D Prescription Drug Plans in 2019 for 3992 Plans

Drug	Covered, % (95% CI)^a^	Covered without prior authorization and without step therapy, % (95% CI)	Covered at tiers 1-3, % (95% CI)^b^	Retail price for 30-d supply, median (IQR), $^c^
GLP-1 receptor agonists				
Liraglutide	92.8 (90.8-94.8)	84.3 (81.9-86.8)	89.6 (87.2-92)	942 (931-969)
Semaglutide (injection)	70.8 (67.3-74.3)	65.9 (62.3-69.6)	67.2 (63.6-70.9)	816 (800-839)
Exenatide ER	94.1 (92.8-95.4)	85.2 (83.2-87.3)	70.5 (67.1-73.9)	732 (707-741)
Exenatide	73.9 (70.4-77.4)	57.5 (53.6-61.4)	9.3 (7.8-10.9)	745 (737-774)
Dulaglutide	93.7 (92.4-95.1)	87.4 (85.5-89.3)	85.7 (83.5-87.9)	765 (738-774)
Lixisenatide	3.5 (2-4.9)	3.2 (1.8-4.6)	0 (0-0)	657 (647-658)
SGLT2 inhibitors				
Empagliflozin	98.5 (97.9-99.1)	95.4 (94.3-96.4)	98.3 (97.7-98.9)	504 (498-519)
Canagliflozin	57.4 (53.2-61.7)	53.2 (49.1-57.4)	50.9 (46.8-55)	520 (507-527)
Dapagliflozin	65.8 (62.2-69.3)	63.7 (60.1-67.3)	41.5 (37.3-45.8)	503 (498-518)
Ertugliflozin	6.3 (4.7-8)	5.5 (3.9-7.1)	0.7 (0.4-1)	300 (285-303)

Abbreviations: GLP-1, glucagon-like peptide 1; IQR, interquartile range; SGLT2, sodium-glucose cotransporter 2.

^a^ All percentages weighted by average plan enrollment during quarter 1 of 2019.

^b^ Tier 1 = preferred generic drug, tier 2 = generic, tier 3 = preferred brand name. Higher tiers are typically associated with higher out-of-pocket costs for beneficiaries.

^c^ Median retail drug costs for 30-day supply of most commonly dispensed dose at in-area retail pharmacies, obtained from the pricing table of the Part D formulary files.

We estimated the median annual out-of-pocket costs for Part D beneficiaries not eligible for low-income subsidies for each drug by using 2 approaches: using the 2019 standard Part D benefit design (ie, 25% of the brand-name drug costs during the initial coverage and coverage gap phases and 5% during catastrophic coverage after a deductible of $415), and using plan-specific benefit information from the formulary files to calculate plan estimated out-of-pocket costs (ie, deductible and co-pay/coinsurance were drawn from observed formulary data). All analyses were performed with SAS version 9.4 and R version 3.6.1. This study follows the Strengthening the Reporting of Observational Studies in Epidemiology (STROBE) reporting guideline for cross-sectional studies and was determined to be exempt by the University of Pittsburgh institutional review board.

## Results

Coverage and retail prices for SGLT2 inhibitors and GLP-1 receptor agonists were variable for 3992 Part D plans during quarter 1 2019 (Table). Excluding ertugliflozin and lixisenatide (which were covered by only 6% and 3% of plans, respectively), coverage without prior authorization and without step therapy requirements ranged from 53.2% (95% CI, 49.1%-57.4%) for canagliflozin to 95.4% (95% CI, 94.3%-96.4%) for empagliflozin. Median retail prices for a 30-day supply ranged from $300 (IQR, $285-$303) for ertugliflozin to $942 (IQR, $931-$969) for liraglutide.

Median estimated annual out-of-pocket costs ranged from $1211 (IQR, $1167-$1221) for ertugliflozin to $2447 (IQR, $2441-$2464) for liraglutide with the standard Part D benefit design. In comparison, median out-of-pocket costs with an algorithm based on plan-specific benefits data were lower, ranging from $1097 (IQR, $932-$1271) for empagliflozin to $2080 (IQR, $1771-$2648) for exenatide (Figure).

**Figure.**
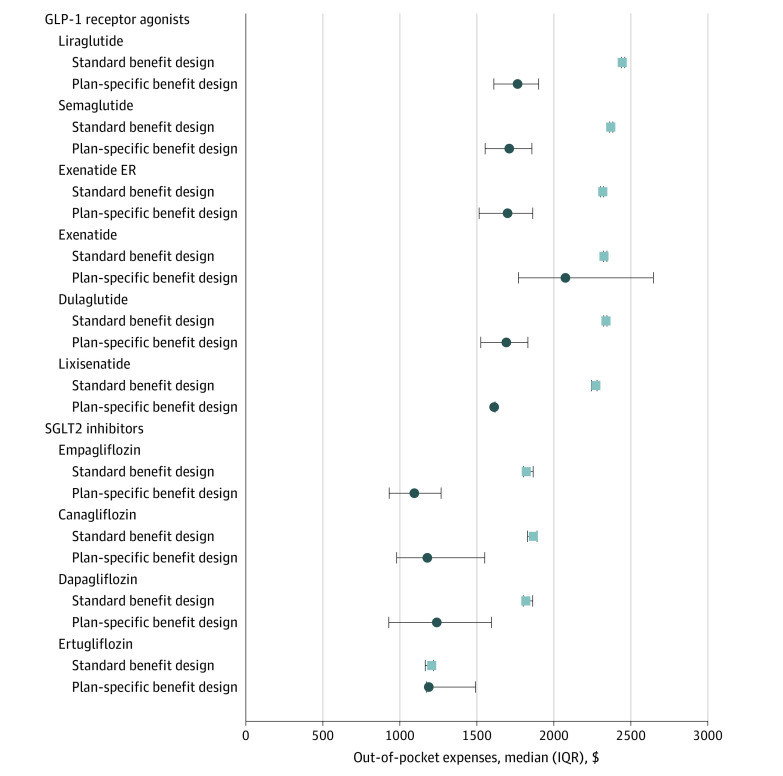
Median Out-of-Pocket Costs for Sodium-Glucose Cotransporter 2 (SGLT2) Inhibitors and Glucagon-Like Peptide 1 (GLP-1) Receptor Agonists Across Medicare Part D in 2019 Using the Standard Benefit Design or an Algorithm Based on Plan-Specific Benefits Data for 3992 Plans In the approach using a standard Medicare Part D benefit design (“standard benefit design”), out-of-pocket costs were calculated with the 2019 benefits structure, in which beneficiaries (not eligible for low-income subsidies) pay 25% of brand-name drug costs (obtained from the pricing file) during both the initial coverage and the coverage gap phases after a deductible of $415. During catastrophic coverage, beneficiaries pay 5% of the brand-name drug cost (or a minimum of $8.50, whichever is greater). In the approach based on plan-specific benefit data, deductible, co-pay, and coinsurance amounts were drawn from plan-specific observations in the formulary files (excluding plans in which exact co-pay or coinsurance amounts could not be directly calculated). Both algorithms assume that a beneficiary fills only 1 drug prescription per month during 12 months. Data are plotted as median (interquartile range).

## Discussion

Coverage for SGLT2 inhibitors and GLP-1 receptor agonists was generally high in 2019 Part D plans, although variable across specific drugs. However, Medicare beneficiaries not eligible for low-income subsidies or Medicaid potentially face very high out-of-pocket costs for SGLT2 inhibitors and GLP-1 receptor agonists. With the exception of less commonly prescribed drugs such as lixisenatide and ertugliflozin, the average beneficiary covered by a Part D plan could spend at least $1000 annually for 1 SGLT2 inhibitor and greater than $1500 for 1 GLP-1 receptor agonist. Although these products are used less frequently than insulin, these annual out-of-pocket costs are on par with—and in some cases exceed—those associated with insulin,^6^ and may be unaffordable for the hundreds of thousands of older adults with diabetes and elevated cardiovascular risk who may receive clinical benefit from one of these newer agents.

Our analysis has 2 main limitations. First, although we weighted our analyses by plan enrollment, we could not account for diabetes-specific enrollment. Second, out-of-pocket cost calculations assumed that beneficiaries only use 1 drug at a time, when in fact older adults using SGLT2 inhibitors and GLP-1 receptor agonists commonly fill other prescriptions, including metformin and insulin. Therefore, our results may overestimate out-of-pocket costs for these drugs among patients who would have reached catastrophic coverage because of simultaneous use of multiple higher-cost drugs. Some beneficiaries may also be insulated from high out-of-pocket costs through patient assistance programs. Although median retail prices do not include proprietary rebates, they do influence the amounts patients pay as coinsurance.
